# Self-perceived functional ability and performance-based testing of physical function in older women with or without long-term back pain – results of the H70 study

**DOI:** 10.1186/s12877-021-02177-y

**Published:** 2021-04-07

**Authors:** Hilda Kristin Svensson, Jon Karlsson, Therese Rydberg Sterner, Felicia Ahlner, Ingmar Skoog, Hanna Falk Erhag

**Affiliations:** 1 Academy of Health and Welfare and Centre of Research on Welfare, Health and Sport (CVHI), Halmstad University, Box 823, SE-301 18 Halmstad, Sweden; 2 Gothenburg Centre for Person-Centred Care (GPCC), Sahlgrenska Academy, University of Gothenburg, Box 457, SE-405 30 Gothenburg, Sweden; 3 Institute of Clinical Sciences and Department of Orthopaedics at Sahlgrenska Academy, University of Gothenburg, Box 426, SE-405 30 Gothenburg, Sweden; 4 Institute of Neuroscience and Physiology at Sahlgrenska Academy, University of Gothenburg, Box 430, SE-405 30 Gothenburg, Sweden; 5 Centre for Ageing and Health (AGECAP) at the University of Gothenburg, Wallinsgatan 6, SE-431 41 Mölndal, Sweden

**Keywords:** Self-rated activities of daily living, Instrumental activities of daily living, Performance-based test of physical function; long-term back pain

## Abstract

**Background:**

The proportion of older adults is increasing rapidly, and the majority are female. In 2050, the number of persons aged 60 years and over is estimated to reach 2.1 billion worldwide, constituting one-third of the total population of Europe. Long-term back pain is a disabling and common condition, primarily affecting older women. Although standardized functional evaluations are essential in the screening of older adults, self-rated activities of daily living capture a different aspect of the person’s ability in the context of his or her environment and social support system. This study aimed to describe how older women with or without long-term back pain self-rate their activities of daily living (ADL) and instrumental activities of daily living (IADL) in relation to their performance-based testing of physical function, including walking test, leg muscle strength, balance, and endurance.

**Method:**

This study is part of the Gothenburg H70 Birth Cohort Studies in Sweden (H70 studies) and uses data from the 1944 birth cohort examined in 2015–16 at age 70. In the present study, only female participants were included in the analysis, and all cases of dementia (*n* = 17) and cases of acute and sub-acute back pain excluded (*n* = 181), leaving an effective sample of 446 female participants.

**Results:**

Women with long-term back pain self-perceived their ADL and IADL as being as good as those without back pain, although they performed poorer in all performance-based tests and perceived themselves as less physically fit.

**Conclusion:**

The discrepancy between self-perceived functional ability (ADL/IADL) and performance-based testing of functioning based on clinical tests calls for further investigation to incentivize person-centered care in older women with long-term back pain in municipal or emergency health-care settings.

## Background

The proportion of older adults is increasing rapidly, and the majority are female. In 2050, the number of persons aged 60 years and over is estimated to reach 2.1 billion worldwide, constituting one-third of the total population of Europe [1, 2]. Accompanying the aging process increases the incidence and prevalence of chronic conditions, functional disability, and symptoms of illness within the proportional changes in the population [3]. Long-term back pain is a common and sometimes disabling condition affecting approximately 23% of older adults, where the majority are female [4]. Compared with men, women report more back pain and experience greater pain-related impairment in daily life [5, 6]. In addition to the female sex, known risk factors for long-term back pain include advancing age, low educational attainment, low physical activity, and being overweight [7]. Among postmenopausal women, the more prominent causes of long-term back pain are age-related degenerative spinal disorders and the compression or fracture of a vertebral body due to osteoporosis [3, 8]. Research shows an association between long-term back pain and reduced quality of life, sense of imprisonment, and social isolation. For several reasons, older women rarely seek treatment for their long-term back pain due to fear of surgery and polypharmacy [9, 10]. It has also been shown that women avoid seeking help for their back pain due to ageist preconceptions, suggesting that long-term back pain is part of normal aging and that health-care professionals will not take them seriously [11, 12].

In clinical practice, back pain can be determined based on the duration of pain after onset, i.e., acute (< 4 weeks), sub-acute (4–12 weeks), and persistent or long-term (> 12 weeks) [13]. Both acute and sub-acute back pain tends to resolve on their own within the first four to six weeks after onset. The vast majority of acute or sub-acute low-back pain is mechanical and associated with overstretching or tearing ligaments, traumatic injury, and herniated or ruptured discs. In long-term back pain, most cases are mainly idiopathic and associated with both normal and pathological changes in the joints, discs, and bones of the spine as people get older. In contrast to acute and sub-acute back pain, long-term back pain shows slight improvement over time, often with remaining functional impairment and functional disability [14–17]. A common way of assessing functional disability is to let people self-rate their ability to perform basic activities of daily living (ADL), including activities such as dressing and feeding, and instrumental activities of daily living (IADL), including activities such as handling finances, grocery shopping and meal preparation [18]. Functional disability increases with age, and among older adults, the proportion rating their ability as impaired differs significantly between age groups and between studies [19]. Performance-based testing of function is another way to determine the ability of older adults. In community-dwelling older adults, different mobility performance tests can predict functional decline greatly and provide helpful information for prognostic assessments [20]. In older acute medical inpatients, physical performance testing such as gait speed, chair stand tests, and balance tests can identify older patients at risk of functional decline and hospital length of stay [21].

Although standardized functional evaluations are important in the screening of older adults, self-rated ADL and IADL capture a different aspect of the person’s ability in the context of his or her environment and social support system. People’s perception of their ability to manage independently in everyday life is of great importance to their quality of life [22, 23]. Little is known about how older women with long-term back pain rate their activities in daily living, instrumental activates of daily living, regardless of their level of physical performance. This study aimed to describe how older women with or without long-term back pain self-rate their ADL and IADL in relation to their performance-based testing of physical function, including walking test, leg muscle strength, balance, and endurance.

## Methods

### Study design and sample

This study is part of the Gothenburg H70 Birth Cohort Studies in Sweden (H70 studies). The H70 studies are multidisciplinary population studies examining representative birth-cohorts of older populations in Gothenburg, Sweden, to study aging and health, taking into account the complex interactions with age, sex, socio-economic gradients, secular changes, as well as psychosocial, neurobiological, and genetic factors occurring across the life course. The studies started in 1971 and combine self-rating, clinically verified health data, and objectively measured indicators. The first cohort of 70-year olds was born in 1901–02, and since then, five waves of new 70-year-olds and three waves of 85-year olds have been examined and followed longitudinally. The examination procedures have been almost identical since the first examination, with a basic examination including a total of six to eight hours of semi-structured interviews with questions about somatic and psychiatric history, psychological and cognitive tests, social factors, functional ability, and physical examination, including blood sampling, ECG, and physiotherapist examination [24].

The present study use data from the 1944 birth cohort examined in 2015–16 at age 70. The participants born in 1944 on pre-specified birth dates and registered as residing in Gothenburg, Sweden, were invited to participate in the study, irrespective of their place of residence (i.e., ordinary or special housing). A total of 1203 persons agreed to participate (response rate 72.2%; 559 men and 644 women; mean age 70.5 years). In the present study, only female participants were included in the analysis, and all cases of dementia (*n* = 17) and cases of acute and sub-acute back pain excluded (*n* = 181), leaving an effective sample of 446 female participants.

### Study procedure

Long-term back pain was ascertained by a positive answer to three questions about the localization, severity, and duration of pain. To be able to detect differences between women with or without long-term back pain, participants were divided into two groups – a target group consisting of women with long-term back pain (*n* = 73) and a reference group comprised of women without back pain (*n* = 373). This study was approved by the Regional Ethical Review Board (registration number 869–13), and informed consent was obtained from all participants.

### Demographics

Educational level was coded as either compulsory education only or at least one more year. Cohabiting included both living with spouse or partner. Height, weight, and BMI were calculated by research nurses at the time of examination. Being able to pursue recreational activities, alcohol consumption, smoking (current and lifetime), falls, use of mobility aids, fractures (hip, femur, wrist, or humerus), vertebral compression fractures, and daily use of analgesics was self-reported.

### ADL and IADL

The Barthel Index of ADL [25] includes ten domains of function (bowels, bladder, grooming, toilet use, feeding, transfer, mobility, dressing, stairs, and bathing). Response options are either independent or dependent/unable and summed to a total score ranging from zero (low function, dependent) to 100 (high function, independent). The Lawton IADL Scale [26] provides information about functional skills necessary to live in the community and includes eight domains of function (ability to use the telephone, shopping, food preparation, housekeeping, laundry, mode of transportation, the responsibility of own medications, and the ability to handle finances). Response options range from fully independent to unable or incapable and are summed to a total score ranging from zero (low function/dependent) to eight (high function/independent). In this study, the self-reported ability was also measured by letting the participants answer questions about whether they considered themselves able to bend down and retrieve something from the floor, able to walk indoors and outdoors and use the stairs. The respondents were also asked to assess their overall fitness level, ranging from physically unfit (poor or very poor) to physically fit (good or very well).

### Performance-based testing of physical function

Walking ability (i.e., self-selected walking speed for 30 m/s), leg muscle strength (i.e., the ability to rise to 50 cm without support), balance (i.e., the ability to stand on one leg with eyes open for 30 s), and endurance (i.e., distance covered in 6 minutes walking at a normal pace) were recorded for all participants by a physiotherapist.

### Statistical analysis

Pearson’s chi-square was used to test differences in proportions. The Mann-Whitney *U*-test was used to test differences in means, while Fisher’s exact test was used for dichotomous variables. IBM SPSS Statistics Data Editor (Version 24) was used for all analyses, and *p*-values of < 0.05 (two-tailed) were considered statistically significant.

## Results

### Participant characteristics

The characteristics of the sample are presented in Table 1. The women with long-term back pain were heavier and had a higher body mass index (BMI) compared to the women without long-term back pain. They also tended to fall more frequently, reported taking more analgesics, and drink less alcohol compared with women without long-term back pain. No differences between the groups were found in terms of body height, living conditions, type of housing, or educational level. However, there was a difference in the proportion reporting that they were able to pursue the recreational activities they enjoyed in their spare time, with women with long-term back pain being less able compared to those without.

**Table 1:** General characteristics of the sample

	Women with long-term back pain *n* = 73 (valid %)	Women without long-term back pain *n* = 373 (valid %)
More than compulsory education	59 (81.9)	317 (86.1)
Cohabiting	40 (55.6)	211 (57.3)
Housing – apartment	54 (75.0)	243 (65.9)
Height (cm), mean (SD)	164.2 (6.0)	164.5 (5.9)
Weight (kg), mean (SD)	76.1 (16.4)	68.7 (12.9) **
BMI (kg/m2), mean (SD)	28.2 (5.9)	25.4 (4.7) **
Fallen ≥ three times in the past year	9 (12.3)	13 (3.5) *
Use mobility/walking aid	8 (11.3)	49 (13.4)
Fractures (hip, femur, wrist, humerus)	18 (24.7)	90 (24.1)
Vertebral compression fractures (VCF)	1 (1.4)	4 (1.1)
Analgesics (≥ one daily prescription)	48 (66.7)	60 (16.2) **
Lifetime smoker	49 (67.1)	219 (58.9)
Drink alcohol never or rarely	30 (44.1)	110 (30.3) *
Able to perform recreational activities that I have reason to value	58 (85.3)	348 (96.7) **

Pearson’s chi-square was used to test differences in proportions, the Mann-Whitney U-test to test differences in means, and Fisher’s exact test was used for dichotomous variables.

*p < 0.05, **p < 0.001

### Self-perceived functional ability and performance-based testing of physical function

There was no difference in self-perceived functional ability between the women with and without long-term back pain except in the ability to climb stairs (Table 2). In both groups, the majority rated themselves as unimpaired in ADL and IADL. There was also a difference in any ADL or IADL problem, where women with long-term back pain were almost four times more likely to have any problem compared to those without pain. There was a large difference in the proportion rating their overall fitness as good or very good, with only 49.3% of the women with long-term back pain rating their fitness as good or very good compared to 83.1% in those without pain. In the performance-based testing of physical function (i.e., walking ability, endurance, leg muscle strength, and balance), the women with long-term back pain performed poorer compared to those without in all tests.

**Table 2:** Self-perceived functional ability and performance-based testing of physical function

	Women with long-term back pain *n* = 73 (valid %)	Women without long-term back pain *n* = 373 (valid %)
Able to bend down	70 (98.6)	354 (96.5)
Able to walk outdoors	67 (94.4)	354 (96.7)
Able to walk indoors	69 (97.2)	359 (97.8)
Able to go for walks without support	65 (91.5)	354 (96.5)
Able to climb stairs without difficulty	62 (87.3)	355 (96.7) *
Assess their own physical fitness as good or very good	35 (49.3)	300 (83.1) **
Barthel index score (ADL), mean (SD)	97.7 (8.3)	97.6 (5.4)
Lawton scale score (IADL), mean (SD)	7.8 (0.7)	7.9 (0.7)
Any ADL or IADL problem	9 (12.3)	13 (3.5) **
Walking ability (self-selected speed) meter/s, mean (SD)	1.3 (0.2)	1.2 (0.2) **
Endurance (distance covered in six minutes walking at a normal pace in meters), mean (SD)	451.9 (106)	529.6 (250.8) **
Leg muscle strength (the ability to rise to 50 cm without support), n (%)	16 (21.9)	134 (35.9) *
Balance (the ability to stand on one leg with eyes open for 30 s), mean (SD)	34 (47.9)	265 (74.0) **

Pearson’s chi-square was used to test differences in proportions, the Mann-Whitney U-test to test differences in means, and Fisher’s exact test was used for dichotomous variables.

*p < 0.05, **p < 0.001

## Discussion

This study showed that there was no difference in self-perceived functional ability between women with long-term back pain and those without long-term back pain, although those with pain did poorer in all performance-based tests of physical function. The women with long-term back pain also perceived themselves as less physically fit compared to those without pain. The discrepancy between self-perceived ability and objectively observed function may have several reasons [27]. Kamitani et al. (2019) found that the discrepancy between high performance-based measurements and low self-reported physical functioning level is associated with an increased risk of future falls in older adults [28]. It is worth considering that the reverse conditions, i.e., low performance-based measurements and high self-reported physical functioning and could also be an indicator of an increased risk of falls in that the person becomes overconfident in her abilities, resulting in carelessness in physical activities. In the study by Kamitani et al. (2019), there were both male and female participants, and it might be worth considering the possibility that there might be a difference between male and female self-reported physical functioning based on gender-labeled traits and preconceptions [28]. It has also been reported that older women tend to both overestimate and underestimate limitations, especially in upper body functions, but the extent to which this could explain discrepancies needs to be further investigated [29]. Beauchamp and colleagues [30] have shown a comparable psychometric property between an interview-administered questionnaire and standardized performance-based measurements, such as gait speed and a 400-m walk and stair climb power test, suggesting the validity of using either of the measurement procedures. Subjective measures may be inaccurate because a person may overestimate or underestimate her capabilities for various reasons. Although objective measurements, such as performance-based tests, can be standardized and objectively scored, they are neither superior to nor interchangeable with subjective measures [31, 32]. Walking speed, for example, has a well-documented predictive value for major health-related outcomes such as hospitalizations, nursing home placements, mortality, poor quality of life, physical and cognitive functional decline, and falls [33]. Assessment of physical function can be of importance both from the perspective of identifying older adults at risk of such events and identifying resources crucial to successful rehabilitation and care [34]. Self-reported outcome measurements provide a person’s perception of her ability and therefore depict a more person-centered assessment, with the potential to estimate the impact on the person’s daily life. On the other hand, the standardized performance-based measurements capture a more objective picture of the current status and might therefore be seen as being a more valid and sensitive estimate of change [19, 29]. According to our knowledge, research is lacking about older women with long-term back pain and how they perceive their ability. In the present study, the women living with long-term back pain reported that they managed and the women without long-term back pain in terms of walking indoors and outdoors, bending down, or any restriction within their home environment in their immediate surroundings. Also, there were no differences between the groups in self-reported ADL and IADL. One important distinction between the use of self-reported outcome measurements and the standardized performance-based test is that the self-reported assessments provide a broad array of functional abilities that are significant for older individuals, while the performance-based tests produce results for a limited number of abilities, such as walking speed and chair standing [33, 34]. This raises the question of what is important within their socio-cultural context and, as a result, what they use in their everyday life and have reason to value. The authors underline the fact that the choice of measurement strategies must be guided by the research question of interest, the complexity and the nature of the data, as well as an awareness that different clinical settings, populations, conditions (e.g., pain, depression, and fear of falling) and contexts are thought to affect the measurements [27, 34]. The use of both performance-based tests of physical function and self-reported functional ability level would create opportunities to identify discrepancies and therefore be able to establish prerequisites for person-centered practice in future care needs [35]. It can be argued that, by assessing both subjective and objective ability, health-care providers might be able both to develop more effective fall prevention plans and to improve the subjective level of assessment, as well as being able to identify persons at risk of adverse events such as injuring falls and early signs of functional decline [28, 34]. This could then lay the foundations for person-centered practice, which, according to the literature, might empower individuals to manage their life situation [35], reinforce and strengthen a sense of security to enhance their quality of life, and continue being resilient, resourceful, and confident in order to find new ways of managing their future everyday life [11, 22, 36, 37].

### Strength and limitations

This study’s strengths include the comprehensive examinations, the large representative population-based sample, and the high response rate (72.3%). There are also some limitations. First, this study used cross-sectional data from one birth cohort of 70-year-old. Using comparable data from other birth cohorts of 70-year-old and cohorts of older ages could have provided insight into cohort differences in self-rated ability and standardized functional evaluations change over time and age in older women since this is a research area that needs development and assiduity. Second, even though the response rate was satisfactory, the possibility cannot be excluded that those who declined participation had poorer health status, including back pain, compared to those who agreed to participate. Third, due to the lack of detailed questions, the definition of long-term back pain used in this study might many potential causes of pain. Fourth, both groups of women self-reported their ADL and IADL as largely unimpaired, which resulted in a ceiling effect (i.e., a large proportion will score at the top of the scale) that limited the range that could be captured.

## Conclusion and implications of key findings

The discrepancy between self-rated functional ability and the results of performance-based tests of physical function in women with long-term back pain is the main finding in this study. This calls for further investigation in order to identify possible underlying individual determinants and explanatory traits in order to develop future interventions in older women with long-term back pain in municipal or emergency health-care settings.

## Data Availability

Due to confidentiality restrictions in the ethical approval of this study, de-identified data may be available from Professor Ingmar Skoog (ingmar.skoog@neuro.gu.se), Director of the Centre of Ageing and Health (AGECAP) at the University of Gothenburg, in response to a reasonable request.

## Abbreviations

ADL: Activities of daily living
IADL: Instrumental activities of daily living
